# Long-Term Health Outcomes in Children Born to Mothers with Diabetes: A Population-Based Cohort Study

**DOI:** 10.1371/journal.pone.0036727

**Published:** 2012-05-23

**Authors:** Chun S. Wu, Ellen A. Nohr, Bodil H. Bech, Mogens Vestergaard, Jørn Olsen

**Affiliations:** 1 Section for Epidemiology, Department of Public Health, Aarhus University, Aarhus, Denmark; 2 Research Unit for General Practice, Aarhus University, Aarhus, Denmark; 3 Section for General Practice, Department of Public Health, Aarhus University, Aarhus, Denmark; 4 Department of Epidemiology, School of Public Health, University of California Los Angeles, Los Angeles, California, United States of America; McGill University, Canada

## Abstract

**Background:**

To examine whether prenatal exposure to parental type 1 diabetes, type 2 diabetes, or gestational diabetes is associated with an increased risk of malignant neoplasm or diseases of the circulatory system in the offspring.

**Methods/Principal Findings:**

We conducted a population-based cohort study of 1,781,576 singletons born in Denmark from 1977 to 2008. Children were followed for up to 30 years from the day of birth until the onset of the outcomes under study, death, emigration, or December 31, 2009, whichever came first. We used Cox proportional hazards model to estimate hazard ratios (HR) with 95% confidence intervals (95% CI) for the outcomes under study while adjusting for potential confounders. An increased risk of malignant neoplasm was found in children prenatally exposed to maternal type 2 diabetes (HR = 2.2, 95%CI: 1.5–3.2). An increased risk of diseases of the circulatory system was found in children exposed to maternal type 1 diabetes (HR = 2.2, 95%CI: 1.6–3.0), type 2 diabetes (HR = 1.4, 95%CI: 1.1–1.7), and gestational diabetes (HR = 1.3, 95%CI: 1.1–1.6), but results were attenuated after excluding children with congenital malformations. An increased risk of diseases of the circulatory system was also found in children exposed to paternal type 2 diabetes (HR = 1.5, 95%CI: 1.1–2.2) and the elevated risk remained after excluding children with congenital malformations.

**Conclusions:**

This study suggests that susceptibility to malignant neoplasm is modified partly by fetal programming. Diseases of the circulatory system may be modified by genetic factors, other time-stable family factors, or fetal programming.

## Introduction

The number of pregnant women with diabetes is increasing as a result of an increasing prevalence of diabetes, a higher age at the time of birth, and earlier onset of diabetes. [1], [2] The health consequences for their children are only partly known. Children born to mothers with diabetes have increased perinatal morbidity and mortality, [3], [4] and more often have early markers of cardiovascular disease [5]–[7] such as insulin resistance. [6] They have a higher birth weight [8] which has been associated with an increased risk of a variety of cancers.[9]–[12] We hypothesized that mothers with diabetes may have children with an increased susceptibility to malignant neoplasm and disease of the circulatory system. Both genetic factors as well as the diabetic intrauterine environment shape disease susceptibility in the offspring. [13].

We therefore conducted a population-based cohort study of 1.7 million children who were followed for up to 30 years to examine whether children born to mothers or fathers with diabetes are more susceptible to malignant neoplasms or diseases of the circulatory system. If parental diabetes affects the outcomes in the offspring mainly through changes in the intrauterine environment, one would expect only maternal diabetes but not paternal diabetes to be associated with the outcomes. If mainly time stable family conditions including genetic factors play a role, one would expect to see both maternal diabetes and paternal diabetes to be associated with the outcomes.

## Methods

### Ethics Statement

According to Danish laws, register-based studies do not need to obtain consents from individuals, when personal identifiers have been encrypted and stored by a trusted third part (Statistic Denmark). The data recruitment was approved by the Danish Data Protection Agency (J.nr.2008-41-2680).

### Study Design, Participants, Exposure, and Outcomes

We identified 1,927,343 singletons born in Denmark from 1977 to 2008 in the Danish Medical Birth Register which includes all births in Denmark since 1973. [14] All persons living in Denmark are assigned a unique personal identification number (civil registration number), which enables accurate linkage of personal data at the individual level. The Danish Civil Registration System was established in 1968 and provides not only civil registry numbers, but also data on vital status and family structure. [15].

The Danish National Hospital Register holds nationwide data on all admissions to any Danish hospital since 1977 and on all outpatient visits since 1995. The information on diabetes was based on the Danish version of the 8^th^ revision of the International Classification of Diseases (ICD-8) from 1977 to 1993, and the 10^th^ revision (ICD-10) from 1994 onwards. [16] All patients with type 1 diabetes or type 2 diabetes were recoded with the same code (ICD-8: 250) between 1977 and 1986 in the Danish version of ICD-8. From 1987 to 1993, the Danish version of ICD-8 differentiated between type 1 diabetes (ICD-8: 249) and type 2 diabetes (ICD-8∶250). From 1994 to 2008, the ICD-10 differentiated between type 1 diabetes (ICD-10: E10, O240), type 2 diabetes (ICD-10: E11, O241), gestational diabetes (ICD-10: O24·4, O24·9), and unspecified diabetes (ICD-10: E12, E13, E14, O242, and O243). We categorized diabetes as unspecified diabetes if the same person were recorded with two or more different ICD codes of diabetes at the same time. If the same person was recorded with different types of diabetes at different times, we categorized diabetes and starting time according to the first ICD code.

We also extracted information on diabetes from the Danish National Diabetes Register that aims at including persons with diabetes who were only treated outside the hospital. The Danish National Diabetes Register holds information on diabetes in the Danish population from 1 January 1995 to 1 January 2007 and it is based on existing registers including the Danish National Hospital Register, the National Health Insurance Service Registry, and the Register of Medicinal Product Statistics. [1], [17] The Danish National Diabetes Register, however, does not differentiate between type 1 diabetes and type 2 diabetes and the register does not include gestational diabetes. [1], [17] We assume that persons with diabetes entirely treated outside the hospital were more likely to have type 2 diabetes, thus any additional diabetes from the Danish National Diabetes Register were categorized as type 2 diabetes.

Children were classified as prenatally exposed to maternal (or paternal) diabetes if they were born after the time when their mothers (or fathers) were recorded with diabetes. Firstly, exposed children born from 1987 to 1993 were categorized as exposed to type 1 or type 2 diabetes at the time of birth. Secondly, Exposed children born from 1994 to 2008 were categorized as exposed to type 1, type 2, or gestational diabetes at the time of birth. Finally, any types of diabetes were grouped in a single category and all exposed children born from 1977 to 2008 and were categorized as exposed to maternal (or paternal) diabetes regardless of types of diabetes.

Information on the outcomes of interest was obtained from the Danish National Hospital Register. Malignant neoplasm were identified as hospitalizations with ICD-8 (140–209) and ICD-10 (C00–C97), diseases of the circulatory system were identified as hospitalizations with ICD-8 (390–458) and ICD-10 (I00–I99), and congenital malformations were identified as hospitalizations with ICD-8 (740-759) and ICD-10 (Q00–Q99).

Information on gestational age, birth weight, maternal age at birth, and parity was obtained from the Danish Medical Birth Registry. [14] Information on maternal education and marital status at the time of birth from 1980 through 2007 was obtained from Statistics Denmark. We used data from 1980 to substitute missing values on maternal education and marital status from 1977 to 1979 and data from 2007 to substitute missing values in 2008, which were not yet available at the time of the study. Some of other missing values on maternal education (or marital status) were replaced by available information in the closest preceding or following five (or three years), whichever came first.

### Statistical Analysis

The children were followed from the day of birth until the first hospitalization or first outpatient visit for the outcomes under study, death, emigration, or December 31, 2009, whichever came first.

Since some children were born to the same mothers, we used robust inference for the Cox proportional hazards model to estimate hazard ratios (HRs) with 95% confidence interval (95% CI) for hospitalization due to malignant neoplasm, diseases of the circulatory system, or congenital malformation for children exposed to maternal or paternal diabetes compared to the cohort of unexposed children. In Model 1, we adjusted for maternal age (five years interval), parity (1, 2, 3+), sex of children (boy, girl), maternal education (low, middle, and high), maternal marital status (yes, no), and calendar year (1987–1990, 1991–1993, 1994–1998, 1999–2003, and 2004–2008). In Model 2, we adjusted for the same variables but excluding children with any congenital malformations. In Model 3, we not only excluded children with any congenital malformations but also extended the adjustment to include gestational age in weeks, birth weight, and birth weight squared as continuous variables.

The statistical analyses were done using Stata11 (StataCorp, College station, TX, USA).

## Results

**Figure 1:**
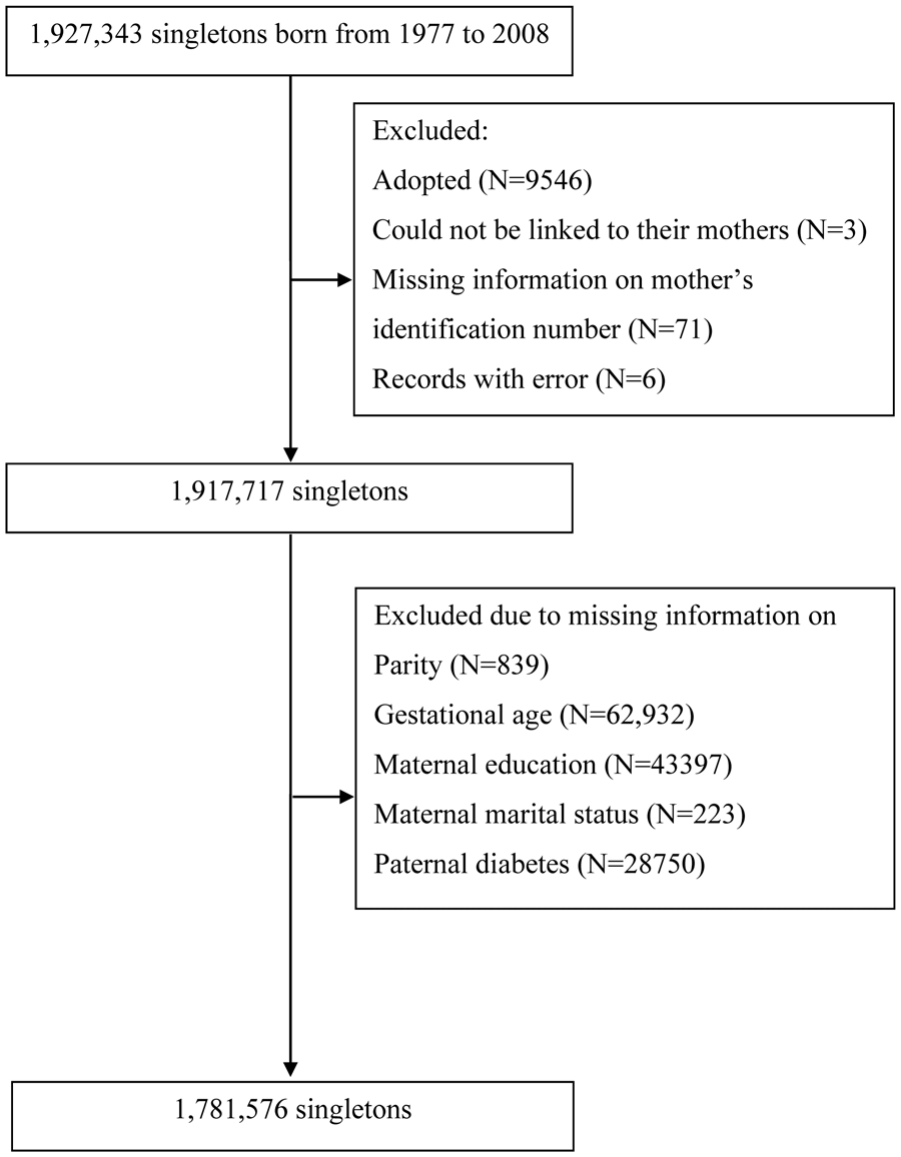
Selection of the study population.

In the final study population, children with missing values on parity, gestational age, birth weight, maternal education, parental marital status, or paternal diabetes were excluded (Figure 1) and 1,781,576 children remained. In the study population, 1,734 (0.1%), 12,401 (0.7%), and 11,507 (0.7%) singletons were prenatally exposed to maternal type 1 diabetes, type 2 diabetes, and gestational diabetes, respectively (Table 1). Pregnant women with type 2 diabetes or gestational diabetes tended to be older than pregnant women without diabetes. A higher proportion of children exposed to maternal diabetes were born preterm (Table 1). Mean birth weight was higher in children exposed to maternal type 1 diabetes (3574 g), type 2 diabetes (3568 g), and gestational diabetes (3583 g) compared to that of children born to mothers without diabetes (3486 g). Children were followed for 15 years on average and up to 30 years.

The prevalence of congenital malformation was increased in singletons exposed to maternal type 1 diabetes, maternal type 2 diabetes, and gestational diabetes when compared to unexposed singletons among singletons born from 1987 to 2008 (Table 2). This increased prevalence was neither seen in singletons exposed to paternal type 1 diabetes nor in singletons exposed to paternal type 2 diabetes (Table 3). The risks did not change much, when analyses were done among all singletons born from 1977 to 2008 and any types of maternal or paternal diabetes were grouped into a single category (Table 2 and 3).

**Table 1:** Characteristics of the study population.

Exposure	Unexposed		TYPE 1 DIABETES		TYPE 2 DIABETES		GESTATIONAL DIABETES	
	N	%	N	%	N	%	N	%
	1,755,712	98.6	1,743	0.1	12,401	0.7	11,507	0.7
**Gender**								
Boy	901,560	51.4	912	52.3	6,354	51.2	5,992	52.1
Girl	854,152	48.7	831	47.7	6,047	48.8	5,515	47.9
**Maternal age**								
<20	43,122	2.5	29	1.7	168	1.4	50	0.4
20–24	340,376	19.4	243	13.9	1,792	14.5	777	6.8
25–29	671,642	38.3	673	38.6	4,166	33.6	3,008	26.1
30–34	502,165	28.6	553	31.7	4,000	32.3	4,283	37.2
35–39	172,677	9.8	214	12.3	1,834	14.8	2,676	23.3
> = 40	25,730	1.5	31	1.8	441	3.6	713	6.2
**Parity**								
1	805,992	45.9	808	46.4	4,298	34.7	4,093	35.6
2	671,117	38.2	651	37.4	5,040	40.6	4,239	36.8
3+	278,603	15.9	284	16.3	3,063	24.7	3,175	27.6
**Gestational week**								
<32	15,570	0.9	68	3.9	263	2.1	141	1.2
33–36	62,296	3.6	449	25.8	1,549	12.5	862	7.5
37	68,865	3.9	522	30.0	1,874	15.1	1,286	11.2
38	180,318	10.3	466	26.7	2,116	17.1	2,748	23.9
39	343,743	19.6	164	9.4	2,078	16.8	3,023	26.3
40	620,869	35.4	53	3.0	2,582	20.8	2,672	23.2
41	319,005	18.2	17	1.0	1,355	10.9	607	5.3
> = 42	145,046	8.3	4	0.2	584	4.7	168	1.5
**Maternal education**								
Low	638,282	36.4	541	31.0	4,707	38.0	4,273	37.1
Middle	560,558	31.9	626	35.9	4,092	33.0	3,488	30.3
High	556,872	31.7	576	33.1	3,602	29.1	3,746	32.6
**Marital status**								
Married	1,081,838	61.6	981	56.3	7,930	64.0	7,538	65.5
Others	673,874	38.4	762	43.7	4,471	36.1	3,969	34.5
**Calendar year**								
1977–1981	220,649	12.6	0	0.0	1,127	9.1	0	0.0
1982–1986	243,977	13.9	0	0.0	1,585	12.8	0	0.0
1987–1990	223,008	12.7	96	5.5	1,287	10.4	0	0.0
1991–1993	183,333	10.4	155	8.9	890	7.2	3	0.0
1994–1998	306,180	17.4	339	19.5	2,125	17.1	2,363	20.5
1999–2003	294,896	16.8	510	29.3	2,727	22.0	3,202	27.8
2004–2008	283,669	16.2	643	36.9	2,660	21.5	5,939	51.6
**Paternal diabetes**								
Unexposed	1,745,515	99.4	1,726	99.0	12,264	98.9	11,314	98.3
TYPE 1 DIABETES	2,971	0.2	3	0.2	27	0.2	46	0.4
TYPE 2 DIABETES	7,039	0.4	14	0.8	108	0.9	139	1.2

**Table 2:** Hazard ratios (HRs) for different health outcomes according to types of maternal diabetes.

	All singletons					Singletons with congenital malformations excluded			
	Person-years (PY)	Number of cases	IR/10^3^ PY	Crude HRs	Model 1^a^	Number of cases	IR/10^3^ PY	Model 2^b^	Model 3^c^
**Maternal diabetes from 1987 to 2008**									
**Malignant neoplasm**									
Unexposed	16,835,352	3,318	0.20			2,707	0.18		
Type 1 diabetes	16,619	4	0.24	1.2	1.3 (0.5–3.5)	3	0.22	1.3 (0.4–4.2)	1.2 (0.4–3.9)
Type 2 diabetes	68,091	27	0.40	2·0	2.2 (1.5–3.2)	19	0.31	1.9 (1.2–3.1)	1.9 (1.2–3.0)
Gestational diabetes	85,308	10	0.12	0.6	0.7 (0.4–1.3)	8	0.10	0.7 (0.3–1.4)	0.7 (0.3–1.3)
**Diseases of the circulatory system**									
Unexposed	16,743,165	19,235	1.15			14,107	0.93		
Type 1 diabetes	16,377	37	2.26	2.3	2.2 (1.6–3.0)	15	1.09	1.4 (0.8–2.3)	1.2 (0.7–2.1)
Type 2 diabetes	67,590	98	1.45	1.5	1.4 (1.1–1.7)	60	0.99	1.3 (1.0–1.6)	1.2 (0.9–1.6)
Gestational diabetes	84,803	113	1.33	1.5	1·3 (1.1–1.6)	70	0.92	1.3 (1.0–1.6)	1.2 (1.0–1.6)
**Congenital malformation**									
Unexposed	15,655,126	121,737	7.78						
Type 1 diabetes	14,455	251	17.36	1.8	1.7 (1·5–2·0)				
Type 2 diabetes	62,765	680	10.83	1.2	1.6 (1·1–1·3)				
Gestational diabetes	78,523	1,125	14.33	1.2	1.2 (1·2–1·3)				
**Any maternal diabetes (1977–2008)**									
**Malignant neoplasm** ^2^									
Unexposed	28,751,460	7,224	0.25			5,935	0.23		
All diabetes	283,573	90	0.32	1.4	1.5 (1.2–1·8)	65	0.26	1.4 (1.1–1.7)	1.3 (1.0–1.7)
**Diseases of the circulatory system**									
Unexposed	28,556,453	41,143	1.44			31,994	1.25		
All diabetes	280,922	484	1.72	1.5	1.4 (1.3–1.5)	305	1.23	1.2 (1.1–1.4)	1.1 (1.0–1.3)
**Congenital malformation**									
Unexposed	26,628,315	171,729	6.45						
All diabetes	256,183	2,911	11.36	1.4	1.3 (1·3–1·4)				

a Model 1: Hazard ratios (HRs) were adjusted for maternal age (<20, 20–24, 25–29, 30–34, 35–39, and 40+), parity (1, 2, and 3+), sex (boy and girl), maternal education (low, middle, and high), maternal marital status (yes or no), calendar year (1977–1981, 1982–1986, 1987–1990, 1991–1993, 1994–1998, 1999–2003, and 2004–2008).

b Model 2: HRs after exclusion of children with congenital malformations and after adjustments for the same variables as that in model 1.

c Model 3: HRs after exclusion o children with congenital malformations and after extended adjustments in the Model 1 by including gestational age at birth as a continuous variable, birth weight, and square of the birth weight.

**Table 3:** Hazard ratios (HRs) for different health outcomes according to types of paternal diabetes.

	All singletons					Singletons with congenital malformation excluded			
	Person-years (PY)	Number of cases	IR/10^3^ PY	Crude HRs	Model 1^a^	Number of cases	IR/10^3^ PY	Model 2^b^	Model 3^c^
**Paternal diabetes were divided into subtypes (1987–2008)**									
**Malignant neoplasm**									
Unexposed	17,167,892	3,422	0.20			2,789	0.18		
Type 1 diabetes	27,915	6	0.21	1.1	1.2 (0.6–2.8)	5	0.20	1.3 (0.5–3.0)	1.3 (0.5–3.0)
Type 2 diabetes	20,123	3	0.15	0.7	0.8 (0.3–2.6)	2	0.11	0.7 (0.2–2.8)	0.7 (0.2–2.8)
**Diseases of the circulatory system**									
Unexposed	17,073,253	19,799	1.16			14,550	0.94		
Type 1 diabetes	27,779	31	1.12	1.2	1.1 (0.8–1.6)	24	0.94	1.2 (0.8–1.9)	1.2 (0.8–1.9)
Type 2 diabetes	19,969	32	1.60	1.7	1.5 (1·1–2·2)	23	1.27	1.6 (1.1–2.5)	1.6 (1.1–2.5)
**Congenital malformation**									
Unexposed	15,964,140	122,947	7.70						
Type 1 diabetes	26,232	245	9.34	1.0	1.0 (0·8–1·1)				
Type 2 diabetes	18,713	228	12.18	1.2	1.1 (1·0–1·3)				
**Any paternal diabetes (1977–2008)**									
**Malignant neoplasm**									
Unexposed	28,751,460	7,224	0.25			5,935	0.23		
All diabetes	131,704	29	0.22	1.0	1.0 (0.7–1.5)	24	0.20	1.0 (0.7–1·5)	1.0 (0.7 -1.5)
**Diseases of the circulatory system**									
Unexposed	28,556,453	41,143	1.44			31,994	1.25		
All diabetes	130,683	191	1.46	1.3	1.2 (1.0–1.4)	149	1.27	1.2 (1.0–1·4)	1.2 (1.0–1.4)
**Congenital malformation**									
Unexposed	26,628,315	171,729	6.45						
All diabees	122,039	973	7.97	1.1	1.0 (1.0–1.1)				

a Model 1: Hazard ratios (HRs) were adjusted for maternal age (<20, 20–24, 25–29, 30–34, 35–39, and 40+), parity (1, 2, and 3+), sex (boy and girl), maternal education (low, middle, and high), maternal marital status (yes or no), calendar year (1977–1981, 1982–1986, 1987–1990, 1991–1993, 1994–1998, 1999–2003, and 2004–2008).

b Model 2: HRs after exclusion of children with congenital malformations and after adjustments for the same variables as that in model 1.

c Model 3: HRs after exclusion of excluding children with congenital malformations and after extended adjustments in the Model 1 by including gestational age at birth as a continuous variable, birth weight, and square of the birth weight.

An increased risk of malignant neoplasm was found in singletons exposed to maternal type 2 diabetes compared to unexposed singletons and the risk remained high after excluding singletons with congenital malformations. The HRs were attenuated a little when analyses were done among all singletons exposed to any types of maternal diabetes (Table 2). A detailed list of ICD codes and number of malignant neoplasm in children exposed to maternal (and paternal) diabetes are available in Table S1.

An increased risk for disease of the circulatory system was found in singletons exposed to maternal type 1 diabetes, maternal type 2 diabetes, and gestational diabetes but these HRs were attenuated when children with congenital malformations were excluded (Table 2). Similar pattern was found when analyses were done among all singletons and any types of maternal or paternal diabetes were grouped into a single category (Table 2). An increased risk for diseases of the circulatory system was also found in singletons exposed to paternal type 2 diabetes. The risks remained high even after excluding children with congenital malformations, but were attenuated when analyses were done among all singletons and any types of maternal or paternal diabetes were grouped into a single category (Table 3). A detailed list of ICD codes and number of disease of the circulatory system in children exposed to maternal (and paternal) diabetes are available in Table S2.

## Discussion

An increased risk for malignant neoplasm was found in children prenatally exposed to maternal type 2 diabetes, and the increased risk remained after excluding children with congenital malformations. An increased risk of disease of the circulatory system was found not only in children exposed to maternal diabetes but also in children exposed to paternal type 2 diabetes.

A higher prevalence of congenital malformations was found in children prenatally exposed to maternal diabetes, which is in line with previous studies. [18]–[23] An increased risk of congenital malformation, however, was not seen in children prenatally exposed to paternal diabetes, which indicates that the observed association between maternal diabetes and congenital malformations may be due to the changes in intrauterine environment induced by maternal diabetes rather than genetic factors.

We found an increased risk of malignant neoplasm in children prenatally exposed to maternal type 2 diabetes and the associations seemed not to be modified by higher birth weight or preterm birth since the results did not change much when we adjusted for these factors. A previous study reported a higher rate of hospitalization due to neoplasms in children born to mothers with diabetes (OR = 1.64, 95%CI: 1.06–2.54) [24] but this was related to maternal insulin dependent diabetes. [25] We did not see an increased risk of malignant neoplasm in children prenatally exposed to paternal diabetes. The results indicate that the risk of malignant neoplasm later in life may to some extent be programmed by a suboptimal intrauterine environment associated with maternal diabetes. The biological mechanism underlying the association is unknown but there are several possible factors that could play a role such as maternal and fetal hyperglycemia [4] and the fetal response to these changes such as hyperinsulinmia. [8] Medical treatments of diabetes during pregnancy may also play a role. Our findings are, however, based on few observations and larger studies with longer follow-up time are wanted. Congenital malformations did not act as a mediator for the observed associations between maternal diabetes and risk of malignant neoplasm since the results did not change much when we excluded children with congenital malformations from the analyses.

Intrauterine exposure to maternal diabetes is associated with childhood overweight, obesity, metabolic syndrome [26], [27], and type 2 diabetes [28] in the offspring, all of which are risk factors for diseases of the circulatory system. An increased risk of hypertension has been reported in offspring of diabetic mothers in humans [6], [29] and animals. [30] We found an overall increased risk of diseases of the circulatory system, not only in children prenatally exposed to maternal diabetes, but also in children exposed to paternal type 2 diabetes, which suggest that genetic or other time-stable family factors also play a role. However, diseases of the circulatory system in our study population were, to large extent, related to congenital abnormalities rather than age-related cardiovascular diseases, which makes congenital malformations act as a mediator for the observed associations between maternal diabetes and risk of disease of the circulatory system. Longer follow-up time is needed for studies on atherosclerosis, myocardial infarction, stroke, and cardiovascular death. In addition, the endpoints in the current study were hospitalizations due to a category of diseases instead of hospitalizations due to a specific disease. Thus the observed associations between maternal or paternal diabetes and disease of the circulatory system may not apply to specific diseases in the categories.

Although type 1 diabetes and type 2 diabetes have common features in terms of increased level of glucose, triglycerides, and many amino acids in the maternal circulation, their genetic background and ability to modify the intrauterine environment probably differ. [31] Previous studies suggest that changes in long-term health outcomes in the offspring of diabetic mothers are not strongly dependent on the type of maternal diabetes, [28], [32], [33] which is supported by some but not all results in the current study.

We used a large population-based cohort including all children born in Denmark and had up to 30 years of almost complete follow-up. The completeness of the registration of diabetes, malignant neoplasm, and diseases of the circulatory system is high since health services, including antenatal care and hospitalizations, are free of charge for all citizens in Denmark. However, some women have type 2 diabetes without knowing it [34] and their children will remain in the unexposed cohort, which would attenuate the associations slightly. In addition, some misclassification between different types of diabetes is likely, [34] especially between 1987 and 1993. We were able to adjust for a number of variables in the analyses but unfortunately, we had no data on lifestyle factors such as maternal smoking, pre-pregnancy body mass index, or breast-feeding. The observed associations could be confounded by these factors.

### Conclusions

This study suggests that susceptibility to malignant neoplasm and congenital malformation may be results of fetal programming induced by maternal diabetes. The risk of disease of circulatory system may be related to genetic factors or other time stable family factors as well as fetal programming. Congenital malformation may act as one of the potential pathways of associations between diabetic intrauterine environment and risk of diseases of the circulatory system in children prenatally exposed to maternal diabetes. We had insufficient follow time to examine risk of atherosclerosis and other aging related cardiovascular diseases. Paternal diabetes had no association with congenital malformation measured as prevalence at birth.

## Supporting Information

Table S1 **A detailed list of the ICD codes and number of malignant neoplasm in children exposed to parental type 1 diabetes (T1D), type 2 diabetes (T2D), and gestational diabetes (GD).**
(DOCX)

Table S2 **A detailed list of the ICD codes and number of disease of circulatory system in children exposed to parental type 1 diabetes (T1D), type 2 diabetes (T2D), and gestational diabetes (GD).**
(DOCX)
