# Supplementary material for: Long-Term Health Outcomes in Children Born to Mothers with Diabetes: A Population-Based Cohort Study
Source: PLoS One. 2012 May 23;7(5):e36727. doi: 10.1371/journal.pone.0036727 (PMC3359312; doi:10.1371/journal.pone.0036727)
Supplement: Table S1 — A detailed list of the ICD codes and number of malignant neoplasm in children exposed to parental type 1 diabetes (T1D), type 2 diabetes (T2D), and gestational diabetes (GD). (DOCX) [file pone.0036727.s001.docx]

Table S1: A detailed list of the ICD codes and number of malignant neoplasm in children exposed to parental type 1 diabetes (T1D), type 2 diabetes (T2D), and gestational diabetes (GD)

| ICD codes | Disease categories | Number of malignant neoplasm in children  exposed to maternal diabetes | | | | | | |  | Number of malignant neoplasm in children  exposed to paternal diabetes | | | | |
| --- | --- | --- | --- | --- | --- | --- | --- | --- | --- | --- | --- | --- | --- | --- |
|  |  | Before exclusion | | |  | After exclusion | | |  | Before exclusion | |  | After exclusion | |
|  |  | T1D | T2D | GD |  | T1D | T2D | GD |  | T1D | T2D |  | T1D | T2D |
| **Malignant neoplasms of digestive organs** | |  |  |  |  |  |  |  |  |  |  |  |  |  |
| C22 | Malignant neoplasm of liver and intrahepatic bile ducts |  | 1 | 1 |  |  | 1 | 1 |  |  |  |  |  |  |
| **Malignant neoplasms of respiratory and intrathoracic organs** | |  |  |  |  |  |  |  |  |  |  |  |  |  |
| C39 | Malignant neoplasm of other and ill-defined sites in the respiratory system and intrathoracic organs |  |  |  |  |  |  |  |  | 1 |  |  | 1 |  |
| **Malignant neoplasms of bone and articular cartilage** | |  |  |  |  |  |  |  |  |  |  |  |  |  |
| C40 | Malignant neoplasm of bone and articular cartilage of limbs |  | 1 |  |  |  | 1 |  |  |  |  |  |  |  |
| C41 | Malignant neoplasm of bone and articular cartilage of other and unspecified sites |  | 3 |  |  |  | 2 |  |  |  |  |  |  |  |
| **Melanoma and other malignant neoplasms of skin** | |  |  |  |  |  |  |  |  |  |  |  |  |  |
| C44 | Other malignant neoplasms of skin |  |  | 1 |  |  |  | 1 |  |  |  |  |  |  |
| **Malignant neoplasms of mesothelial and soft tissue** | |  |  |  |  |  |  |  |  |  |  |  |  |  |
| C47 | Malignant neoplasm of peripheral nerves and autonomic nervous system |  |  | 1 |  |  |  | 1 |  | 1 |  |  |  |  |
| C49 | Malignant neoplasm of other connective and soft tissue |  | 1 |  |  |  | 1 |  |  |  |  |  |  |  |
| **Malignant neoplasms of female genital organs** | |  |  |  |  |  |  |  |  |  |  |  |  |  |
| C53 | Malignant neoplasm of cervix uteri |  | 1 |  |  |  | 1 |  |  |  |  |  |  |  |
| C56 | Malignant neoplasm of ovary |  |  |  |  |  |  |  |  | 1 |  |  | 1 |  |
| **Malignant neoplasms of male genital organs** | |  |  |  |  |  |  |  |  |  |  |  |  |  |
| 186 | Malignant neoplasm of testis |  | 1 |  |  |  | 1 |  |  |  |  |  |  |  |
| **Malignant neoplasms of urinary tract** | |  |  |  |  |  |  |  |  |  |  |  |  |  |
| C64 | Malignant neoplasm of kidney, except renal pelvis |  | 3 | 1 |  |  | 1 | 1 |  |  | 1 |  | 0 | 1 |
| C67 | Malignant neoplasm of bladder |  | 1 |  |  |  | 1 |  |  |  |  |  |  |  |
| **Malignant neoplasms of eye, brain and other parts of central nervous system** | | | | |  |  |  |  |  |  |  |  |  |  |
| C69 | Malignant neoplasm of eye and adnexa |  | 2 | 2 |  |  | 1 | 1 |  |  | 1 |  | 0 | 1 |
| 191 | Malignant neoplasm of brain |  | 1 | 0 |  |  | 1 |  |  |  |  |  |  |  |
| C71 | Malignant neoplasm of brain | 1 | 2 | 1 |  |  | 2 | 1 |  |  |  |  |  |  |
| **Malignant neoplasms of ill-defined, secondary and unspecified sites** | |  |  |  |  |  |  |  |  |  |  |  |  |  |
| C76 | Malignant neoplasm of other and ill-defined sites |  | 1 |  |  |  | 1 |  |  |  |  |  |  |  |
| **Malignant neoplasms, stated or presumed to be primary, of lymphoid, haematopoietic and related tissue** | | | | | | | | |  |  |  |  |  |  |
| 208 | Polycythaemia vera | 1 | 2 |  |  | 1 | 1 |  |  | 2 |  |  | 2 |  |
| C91 | Lymphoid leukaemia | 1 | 6 | 1 |  | 1 | 4 | 1 |  |  | 1 |  |  |  |
| C92 | Myeloid leukaemia |  | 1 | 1 |  |  |  | 1 |  |  |  |  |  |  |
| C94 | Other leukaemias of specified cell type |  |  | 1 |  |  |  |  |  |  |  |  |  |  |
| C95 | Leukaemia of unspecified cell type | 1 |  |  |  | 1 |  |  |  | 1 |  |  | 1 |  |
|  |  |  |  |  |  |  |  |  |  |  |  |  |  |  |
| Total | | 4 | 27 | 10 |  | 3 | 19 | 8 |  | 6 | 3 |  | 5 | 2 |
