# Supplementary material for: Long-Term Health Outcomes in Children Born to Mothers with Diabetes: A Population-Based Cohort Study
Source: PLoS One. 2012 May 23;7(5):e36727. doi: 10.1371/journal.pone.0036727 (PMC3359312; doi:10.1371/journal.pone.0036727)
Supplement: Table S2 — A detailed list of the ICD codes and number of disease of circulatory system in children exposed to parental type 1 diabetes (T1D), type 2 diabetes (T2D), and gestational diabetes (GD). (DOCX) [file pone.0036727.s002.docx]

Table S2: A detailed list of the ICD codes and number of disease of circulatory system in children exposed to parental type 1 diabetes (T1D), type 2 diabetes (T2D), and gestational diabetes (GD)

| ICD codes | Disease categories | Number of disease of circulatory system  in children exposed to maternal diabetes | | | | | | |  | Number of disease of circulatory system in children exposed to maternal diabetes | | | | |
| --- | --- | --- | --- | --- | --- | --- | --- | --- | --- | --- | --- | --- | --- | --- |
|  |  | Before exclusion | | |  | After exclusion | | |  | Before exclusion | |  | After exclusion | |
|  |  | T1D | T2D | GD |  | T1D | T2D | GD |  | T1D | T2D |  | T1D | T2D |
| **Chronic rheumatic heart diseases** | |  |  |  |  |  |  |  |  |  |  |  |  |  |
| 393 | Diseases of pericardium |  | 1 |  |  |  | 1 |  |  |  |  |  |  |  |
| I08 | Multiple valve diseases |  |  |  |  |  |  |  |  | 1 |  |  |  |  |
| **Hypertensive diseases** | |  |  |  |  |  |  |  |  |  |  |  |  |  |
| I10 | Essential (primary) hypertension |  | 1 | 2 |  |  | 1 | 2 |  | 1 | 3 |  | 1 | 2 |
| I12 | Hypertensive renal disease |  |  | 2 |  |  |  | 1 |  |  |  |  |  |  |
| I15 | Secondary hypertension |  | 1 |  |  |  |  |  |  | 1 |  |  | 1 |  |
| **Ischaemic heart diseases** | |  |  |  |  |  |  |  |  |  |  |  |  |  |
| I20 | Angina pectoris |  | 2 |  |  |  | 2 |  |  |  |  |  |  |  |
| I24 | Other acute ischaemic heart diseases |  |  |  |  |  |  |  |  |  | 1 |  |  | 1 |
| **Pulmonary heart disease and diseases of pulmonary circulation** | |  |  |  |  |  |  |  |  |  |  |  |  |  |
| I27 | Other pulmonary heart diseases |  | 1 | 1 |  |  | 1 |  |  | 1 | 1 |  |  |  |
| **Other forms of heart disease** | |  |  |  |  |  |  |  |  |  |  |  |  |  |
| 421 | Acute and subacute endocarditis | 1 | 1 |  |  | 1 |  |  |  |  |  |  |  |  |
| 425 | Cardiomyopathy | 1 | 1 |  |  |  |  |  |  |  |  |  |  |  |
| 426 | Pulmonary heart disease |  | 3 |  |  |  |  |  |  |  |  |  |  |  |
| 427 | Symptomatic heart disease | 1 | 2 |  |  |  | 2 |  |  |  |  |  |  |  |
| 429 | Ill-defined heart disease | 1 | 2 |  |  |  | 1 |  |  |  |  |  |  |  |
| I30 | Acute pericarditis |  | 3 | 1 |  |  | 3 | 1 |  |  |  |  |  |  |
| I31 | Other diseases of pericardium |  |  | 1 |  |  |  |  |  |  | 1 |  |  |  |
| I33 | Acute and sub-acute endocarditis |  |  | 1 |  |  |  | 1 |  |  |  |  |  |  |
| I34 | Nonrheumatic mitral valve disorders |  | 1 | 2 |  |  |  | 1 |  | 1 |  |  |  |  |
| I35 | Nonrheumatic aortic valve disorders |  | 2 | 5 |  |  |  | 1 |  | 1 |  |  | 1 |  |
| I36 | Nonrheumatic tricuspid valve disorders | 1 |  |  |  | 1 |  |  |  |  | 1 |  |  | 1 |
| I37 | Pulmonary valve disorders |  | 2 | 1 |  |  | 1 |  |  |  |  |  |  |  |
| I39 | Endocarditis and heart valve disorders in diseases classified elsewhere | 1 |  |  |  |  |  |  |  | 1 |  |  |  |  |
| I42 | Cardiomyopathy | 7 | 7 | 12 |  | 1 | 3 | 5 |  |  |  |  |  |  |
| I43 | Cardiomyopathy in diseases classified elsewhere | 2 | 1 |  |  | 1 |  |  |  |  |  |  |  |  |
| I44 | Atrioventricular and left bundle-branch block | 1 | 1 | 1 |  |  |  | 1 |  | 3 |  |  | 2 |  |
| I45 | Other conduction disorders | 2 | 1 | 3 |  | 2 |  | 3 |  |  | 1 |  |  | 1 |
| I46 | Cardiac arrest | 1 | 2 | 3 |  | 1 |  | 2 |  |  | 1 |  |  | 1 |
| I47 | Paroxysmal tachycardia | 3 | 12 | 7 |  | 2 | 8 | 5 |  | 2 | 1 |  | 2 | 1 |
| I48 | Atrial fibrillation and flutter |  |  | 1 |  |  |  | 1 |  |  |  |  |  |  |
| I49 | Other cardiac arrhythmias | 2 | 8 | 1 |  |  | 6 | 1 |  | 2 |  |  | 1 |  |
| I50 | Heart failure | 1 | 1 | 5 |  |  |  | 1 |  | 2 | 2 |  | 2 |  |
| I51 | Complications and ill-defined descriptions of heart disease | 2 | 4 | 8 |  | 1 | 1 | 5 |  |  | 1 |  |  | 1 |
| **Cerebrovascular disease** | |  |  |  |  |  |  |  |  |  |  |  |  |  |
| 433 | Cerebral thrombosis |  | 1 |  |  |  |  |  |  |  |  |  |  |  |
| I60 | Subarachnoid haemorrhage |  | 1 | 1 |  |  |  |  |  |  | 1 |  |  | 1 |
| I61 | Intracerebral haemorrhage | 1 |  | 1 |  |  |  | 1 |  |  | 1 |  |  |  |
| I62 | Other nontraumatic intracranial haemorrhage |  |  | 1 |  |  |  | 1 |  |  |  |  |  |  |
| I63 | Cerebral infarction |  | 3 | 3 |  |  | 3 | 3 |  |  |  |  |  |  |
| 164 | Stroke, not specified as haemorrhage or infarction |  |  |  |  |  |  |  |  |  | 1 |  |  |  |
| I65 | Occlusion and stenosis of precerebral arteries, not resulting in cerebral infarction |  |  | 1 |  |  |  | 1 |  |  |  |  |  |  |
| I67 | Other cerebrovascular diseases |  |  | 1 |  |  |  | 1 |  |  | 1 |  |  | 1 |
| I69 | Sequelae of cerebrovascular disease |  | 3 | 2 |  |  | 3 | 1 |  | 1 |  |  | 1 |  |
| **Diseases of arteries, arterioles and capillaries** | |  |  |  |  |  |  |  |  |  |  |  |  |  |
| 443 | Other peripheral vascular disease |  |  |  |  |  |  |  |  | 2 |  |  | 2 |  |
| 445 | Gangrene | 1 |  |  |  |  |  |  |  |  |  |  |  |  |
| I70 | Atherosclerosis | 1 | 1 |  |  |  | 1 |  |  |  |  |  |  |  |
| I71 | Aortic aneurysm and dissection |  |  | 1 |  |  |  |  |  |  |  |  |  |  |
| I72 | Other aneurysm and dissection |  |  | 1 |  |  |  | 1 |  |  |  |  |  |  |
| I73 | Other peripheral vascular diseases |  |  | 4 |  |  |  | 3 |  | 1 | 1 |  | 1 | 1 |
| I74 | Arterial embolism and thrombosis |  | 1 | 1 |  |  | 1 | 1 |  |  |  |  |  |  |
| I77 | Other disorders of arteries and arterioles | 1 | 1 | 2 |  |  | 1 | 1 |  |  |  |  |  |  |
| I78 | Diseases of capillaries |  | 2 | 4 |  |  | 1 | 2 |  |  |  |  |  |  |
| **Diseases of veins, lymphatic vessels and lymph nodes, not elsewhere classified** | | | | |  |  |  |  |  |  |  |  |  |  |
| 455 | Haemorrhoids |  | 1 |  |  |  |  |  |  |  |  |  |  |  |
| 458 | Other diseases of circulatory system |  | 1 |  |  |  |  |  |  |  | 1 |  |  | 1 |
| I80 | Phlebitis and thrombophlebitis |  | 1 |  |  |  | 1 |  |  | 1 | 1 |  | 1 | 1 |
| I82 | Other venous embolism and thrombosis |  | 1 | 1 |  |  | 1 | 1 |  |  | 1 |  |  | 1 |
| I83 | Varicose veins of lower extremities |  | 1 | 1 |  |  |  | 1 |  | 2 |  |  | 2 | 0 |
| I84 | Haemorrhoids | 1 | 4 | 6 |  | 1 | 4 | 5 |  | 2 | 3 |  | 2 | 2 |
| I86 | Varicose veins of other sites |  | 3 | 1 |  |  | 3 | 1 |  |  |  |  |  |  |
| I88 | Nonspecific lymphadenitis | 3 | 6 | 17 |  | 3 | 6 | 12 |  | 4 | 7 |  |  |  |
| I89 | Other noninfective disorders of lymphatic vessels and lymph nodes |  | 1 | 6 |  |  | 1 | 3 |  | 1 |  |  | 4 | 6 |
| **Other and unspecified disorders of the circulatory system** | |  |  |  |  |  |  |  |  |  |  |  |  |  |
| I95 | Hypotension |  | 2 |  |  |  | 1 |  |  |  |  |  |  |  |
| I97 | Postprocedural disorders of circulatory system, not elsewhere classified |  | 1 | 1 |  |  |  |  |  |  |  |  |  |  |
| I98 | Other disorders of circulatory system in diseases classified elsewhere |  | 1 |  |  |  | 1 |  |  |  |  |  |  |  |
| I99 | Other and unspecified disorders of circulatory system | 2 | 2 |  |  | 1 | 2 |  |  | 1 | 1 |  | 1 | 1 |
|  |  |  |  |  |  |  |  |  |  |  |  |  |  |  |
| Total | | 37 | 98 | 113 |  | 15 | 60 | 70 |  | 31 | 32 |  | 24 | 23 |
